# Methylation of *TET2* Promoter Is Associated with Global Hypomethylation and Hypohydroxymethylation in Peripheral Blood Mononuclear Cells of Systemic Lupus Erythematosus Patients

**DOI:** 10.3390/diagnostics12123006

**Published:** 2022-12-01

**Authors:** Wan-Yu Sung, Yuan-Zhao Lin, Daw-Yang Hwang, Chia-Hui Lin, Ruei-Nian Li, Chia-Chun Tseng, Cheng-Chin Wu, Tsan-Teng Ou, Jeng-Hsien Yen

**Affiliations:** 1 Division of Rheumatology, Department of Internal medicine, Kaohsiung Medical University Hospital, Kaohsiung 80756, Taiwan; 2 National Institute of Cancer Research, National Health Research Institutes, Tainan 350401, Taiwan; 3 Division of Nephrology, Department of Internal medicine, Kaohsiung Medical University Hospital, Kaohsiung 80756, Taiwan; 4 Department of Biomedical Science and Environmental Biology, College of Life Science, Kaohsiung Medical University, Kaohsiung 80756, Taiwan; 5 Graduate Institute of Clinical Medicine, College of Medicine, Kaohsiung Medical University, Kaohsiung 80708, Taiwan; 6 College of Biological Science and Technology, National Chiao Tung University, Hsinchu 30010, Taiwan; 7 Institute of Medical Science and Technology, National Sun Yat-Sen University, Kaohsiung 80424, Taiwan

**Keywords:** systemic lupus erythematosus, methylation, hydroxymethylation, TET methylcytosine dioxygenase enzymes, next-generation sequencing

## Abstract

(1) Background: It is widely accepted that aberrant methylation patterns contribute to the development of systemic lupus erythematosus (SLE). Ten–eleven translocation (TET) methylcytosine dioxygenase is an essential enzyme of which there are three members, TET1, 2, and 3, involved in hydroxymethylation, a newly uncovered mechanism of active DNA methylation. The epigenomes of gene transcription are regulated by 5-hydroxymethylcytocine (5-hmC) and TETs, leading to dysregulation of the immune system in SLE. The purpose of this study was to investigate the global hydroxymethylation status in SLE peripheral blood mononuclear cells (PBMCs) and to explore the role of TETs in changing the patterns of methylation. (2) Methods: We collected PBMCs from 101 SLE patients and 100 healthy donors. TaqMan real-time polymerase chain-reaction assay was performed for the detection of 5-methylcytosine (5-mC), 5-hmC, and TET2 mRNA expression and single-nucleotide polymorphism genotyping. The methylation rates in different CpG sites of TET2 promoters were examined using next-generation sequencing-based deep bisulfite sequencing. Putative transcription factors were investigated using the UCSC Genome Browser on the Human Dec. 2013 (GRCh38/hg38) Assembly. (3) Results: 5-mC and 5-hmC were both decreased in SLE. The mRNA expression level of TET2 was notably high and found to be correlated with the levels of immunologic biomarkers that are indicative of SLE disease activity. The analysis of methylation rates in the TET2 promoter revealed that SLE patients had significantly higher and lower rates of methylation in TET2 105146072-154 and TET2 105146218-331, respectively. (4) Conclusions: TET2 may play an important role in 5-mC/5-hmC dynamics in the PBMCs of SLE patients. The epigenetic modification of TET2 promoters could contribute to the pathogenesis of SLE and the intensity of the immunologic reaction.

## 1. Introduction

Systemic lupus erythematosus (SLE) is a prototypical autoimmune disease characterized by hyperactivity of the immune system that results in multiple spontaneous inflammatory responses and subsequent tissue damage and organ failure. The dysregulation of immune responses is currently considered to be related to genes, sex hormones [1,2,3], and environmental factors (such as ultraviolet light, infections, free radicals, and environmental hormones) [4]. One study of homozygotic twins showed that the incidence of incomplete concordance is about 19–59% [5,6,7], which demonstrates that nongenetic factors play a pivotal role in the occurrence of SLE [6,8,9].

Epigenetic mechanisms include DNA methylation, miRNA interactions, and histone modification [10], of which the most important gene-regulation mechanism is DNA methylation. Among the research into nonhereditary causes of SLE, aberrant DNA methylation has attracted the most attention [11,12,13,14,15]. When the gene body loses its normal methylation pattern, the gene becomes abnormally activated, which can lead to dysgenesis, disease development, or survival failure of the organism. Although many studies have found many factors affecting DNA demethylation [12,13,14,15,16,17,18,19,20], it was not until 5-hydroxymethylcytosine (5-hmC) was identified in mammalian cells that there was a significant breakthrough in uncovering how the methyl group was removed from DNA in these cells.

The 5-hmC modification was first observed in the DNA of bacteriophages [21]. In mammals, 5-hmC is a product of 5-methylcytosine (5-mC) hydroxylation and referred to as hydroxymethylation, and it is further oxidized into other cytosine derivatives, 5-formylcytosine and 5-carboxycytosine [22,23,24,25], which are quite unstable and 100-fold less abundant than 5-hmC in the DNA-demethylation pathway [22,26,27].

The stepwise oxidation of 5-mC is catalyzed by ten–eleven translocation (TET) enzymes, which is one demethylation mechanism. TET protease is a family of three members, TET1, TET2, and TET3, which have similar crystallographic structures but whose genes are located on different chromosomes [28]. TETs, as methylcytosine dioxygenases, are indispensable for hydroxymethylation and may even serve as an influencing component of the rate-determination step [29]. Although TETs are observed to have different functions in different cells and tissues [29,30], knowledge of these functions is currently limited. For instance, TET1 and TET2 are highly expressed in mouse embryonic stem cells, whereas TET3 is largely expressed in the egg and single-cell zygote phase [31]. In *TET* knockout studies, it was found that TET1 is related to the body-size development of mice, TET2 is related to abnormal blood-cell function, and embryos lacking TET3 cannot survive or have extremely severe abnormalities [29]. Studies in humans have shown that mutations in *TET2* are thought to be the most relevant of the various TETs in developing hematological malignancies [32,33,34] and are also associated with increased risks of autoimmunity in myelodysplastic syndrome patients [35].

The discovery of hydroxymethylation answered some questions on how methyl groups are lost from DNA and provided a clue for active DNA demethylation. The modification of 5-hmC is considered to be the sixth base in epigenetic modification that regulates DNA methylation patterns, whereas its precursor, 5-mC, is the fifth base [36]. It was found that the pathogenesis of SLE is closely related to aberrant methylation, so in our study, we aim to explore these two effects in SLE peripheral blood mononuclear cells (PBMCs) in addition to the interaction between hydroxymethylation and TET enzymes.

## 2. Materials and Methods

### 2.1. Patients

Samples of peripheral blood were obtained from SLE patients who had been diagnosed according to the 2019 European League Against Rheumatism/American College of Rheumatology Classification Criteria for SLE [37]. These patients were all treated in the outpatient or inpatient department according to the clinical context. The healthy donors were recruited from the healthy population in the community. The process, involving informed consent, was approved by the Institutional Ethical Committee of Kaohsiung Medical University Hospital.

### 2.2. Methods

#### 2.2.1. Isolation of Mononuclear Cells

PBMCs were isolated using Ficoll-Paque PLUS (GE Healthcare Bio-Sciences AB, Concord, MA, USA).

#### 2.2.2. Genomic DNA and RNA Extraction

The extraction of DNA and RNA was performed by using a Genomic DNA Extraction Kit (QIAGEN, Hilden, Germany) and RNA Blood Mini Kit (QIAamp, Ltd., Hilden, Germany), respectively, according to the manufacturer’s instructions.

#### 2.2.3. DNA Methylation and Hydroxymethylation Assay

Global methylation and hydroxymethylation levels were quantified on total DNA using the MethylFlash^TM^ Methylated DNA Quantification Kit (Epigentek, Farmingdale, NY, USA) and MethylFlash^TM^ Hydroxymethylated DNA Quantification Kit (Epigentek, Farmingdale, NY, USA), respectively, according to the manufacturer’s instructions.

#### 2.2.4. mRNA Expression of TETs

The mRNA expression levels of the target genes, *TET1*, *TET2*, and *TET3*, were quantified on RNA using Applied Biosystems TaqMan^®^ Gene Expression Assays (*TET1*, Hs04189341; *TET2*, Hs00758658; *TET3*, Hs00379125). RNA polymerase II was used as an internal reference due to its constant and stable RNA-transcription level under stimulation [38].

#### 2.2.5. Single-Nucleotide Polymorphism (SNP) Genotyping

TaqMan^®^ SNP Genotyping Assays (Applied Biosystems. No. C_33251908, C_2551990, C_26458781) for *TET2* gene polymorphism identification was used. Sequence detection of TaqMan real-time PCRs and genotyping analysis were performed using the ABI Prism 7500 system.

#### 2.2.6. Next-Generation Sequencing (NGS)-Based Deep Bisulfite Sequencing

Deep bisulfite sequencing was performed according to the protocol proposed by Leitão et al. [39]. Bisulfite conversion of DNA was accomplished by using the EpiTect Fast DNA Bisulfite Kit (QIAGEN, Germany). After bisulfite treatment of DNA, PCR was used to amplify different sections of *TET2* promoter using five pairs of forward and reverse primers (Table 1) designed using PyroMark Assay Design SW 2.0 (QIAGEN, Germany). First-round gene-specific PCRs were performed using the HotStarTaq Master Mix Kit (QIAGEN, Germany), which consisted of the initial hot start at 95 °C for 15 min followed by 50 cycles of denaturation at 94 °C for 30 s, annealing at 56 °C for 45 s, extension at 72 °C for 45 s, and a final extension phase at 72 °C for 10 min. The second PCR procedure was performed by using Index Adapters IDT^®^ for Illumina Nextera DNA Unique Dual Indexes Set A (96 Indexes, 96 Samples) (Illumina, San Diego, CA, USA). PCR products were purified with Agencourt AMPure XP Beads (Beckman Coulter, Inc., Pasadena, CA, USA) and quantified using a Qubit^TM^ 1X dsDNA HS Assay Kit (Thermo Fisher Scientific, Waltham, MA, USA). NGS was performed using the MiniSeq Mid Output Kit on the MiniSeq Illumina System (Illumina, USA) platform following the manufacturer’s instructions. The sequencing reaction was performed using 250 base paired-end sequencing. FASTQ files were further analyzed with the CLC Genomics Workbench v.10.0 Bisulfite Sequencing Plugin 2.1 (QIAGEN), which provides detailed nucleotide-level analysis, including the calculation of CpG methylation rates. Subsequent analysis of methylation rates was performed according to the criteria set by Masser et al. [40]. The putative transcription factors were searched from the UCSC Genome Browser on Human Dec. 2013 (GRCh38/hg38) Assembly.

### 2.3. Statistics

Statistical analysis was performed using the SPSS software, version 19, with *t*-testing for comparison of methylation and hydroxymethylation levels in the two populations in addition to methylation rates in various CpG sites of *TET2*. SNPs and the relationships between clinical manifestations and SNPs were analyzed using the chi-square test. Demographic data and *TET1*, *TET2*, and *TET3* mRNA expression were analyzed using one-way analysis of variance (ANOVA). The correlations between the levels of *TET* mRNA vs. anti-dsDNA antibody and complement level vs. each CpG site were analyzed based on Spearman’s correlation. The confounder effect on global methylation, global hydroxymethylation, *TET2* mRNA expression, and DNA methylation level was examined using multivariate linear-regression analysis. Causal-inference analysis was performed to explore the SLE disease status, global methylation/hydroxymethylation, and *TET2* methylation. Statistical significance was defined as *p* < 0.05.

## 3. Results

We collected PBMCs from 101 SLE patients and 100 healthy donors. The demographic information is shown in Table 1.

### 3.1. Global Methylation and Hydroxymethylation

In the PBMCs of SLE and healthy donors, SLE patients had both global hypomethylation (5-mC in SLE and the healthy donors; 1.1093 ± 0.7103 vs. 1.3645 ± 0.8302, respectively; *p* = 0.021) and hypohydroxymethylation (5-hmC in SLE and the healthy donors; 0.0653 ± 0.0980 vs. 0.1091 ± 0.1326, respectively; *p* = 0.012), as shown in Table 2. To evaluate which variables affected the methylation pattern, multivariate regression was performed on age, gender, methotrexate, and cyclophosphamide as potential drivers of the difference in global methylation and hydroxymethylation, and the results are shown in Table 3.

### 3.2. TET mRNA Expression

**Figure 1:**
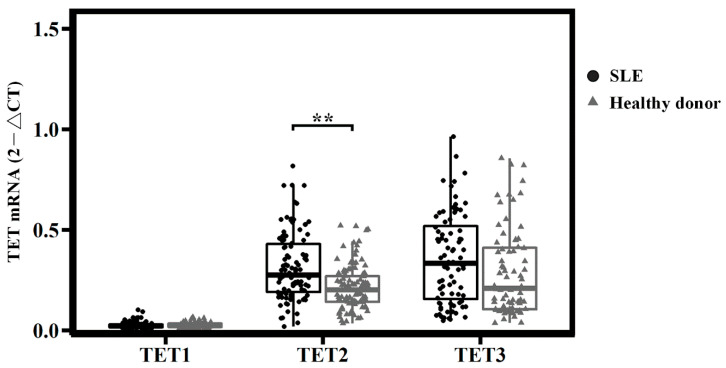
mRNA expression of TETs in systemic lupus erythematosus and healthy donors. *TET2* was remarkably high in SLE, ** *p* < 0.001. *p*-values were obtained using ANOVA tests.

In the healthy donors, the mRNA expression levels of TET genes all differed according to the following order, from highest to lowest: *TET3* > *TET2* > *TET1* (Figure 1); in SLE, the *TET2* and *TET3* expression levels were significantly higher than those of *TET1*. Comparing the expression of every *TET* between SLE and the healthy donors, *TET2* showed remarkably high levels in SLE (0.3293 ± 0.2139 vs. 0.2161 ± 0.1092, respectively; *p* < 0.001); the expressions of *TET1* and *TET3* did not differ between the two groups (Figure 1). The factors of age, gender, and medication were also statistically analyzed to assess the confounder effect on mRNA expression (Table 3).

### 3.3. TET2 mRNA and Immunologic Biomarkers

Immunologic tests of anti-dsDNA antibody and complement levels, as markers of SLE disease activity, are commonly available in the clinical context. An increase in anti-dsDNA antibody levels is a predictor of disease flare, and the complement level is correlated inversely with disease activity [41]. We found that TET2 mRNA expression was correlated with anti-dsDNA titers (r = 0.379, *p* < 0.01) and C3 and C4 levels (r = −0.257 and −0.328, *p* < 0.05 and <0.01, respectively; Table 4).

### 3.4. SNPs of TET2

**Figure 2:**
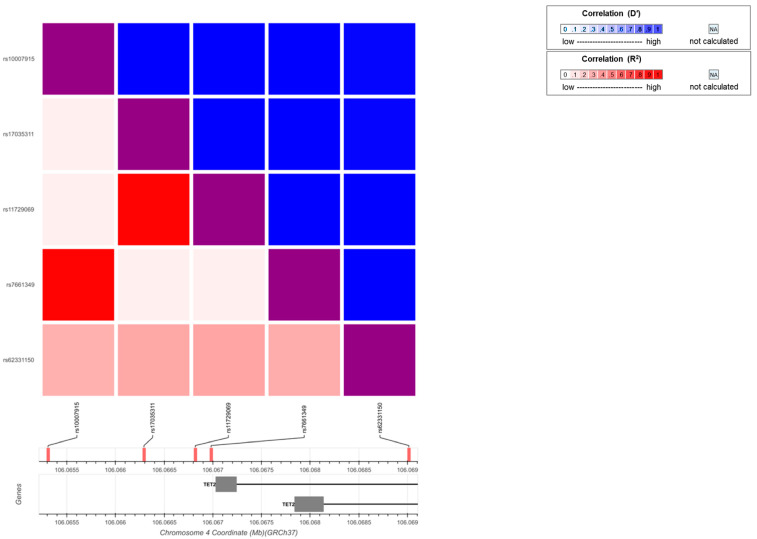
Linkage-disequilibrium plot for *TET2* SNPs. To avoid genetic linkage, which can lead to issues in multiple comparisons, a heatmap was created using an interactive online tool (https://Idlink.nci.nih.gove/?tab=Idmatrix, accessed on 20 September 2020) to understand the patterns of linkage disequilibrium between SNPs.

We inspected whether SNPs counted for the increased expression of *TET2* mRNA in SLE patients. Five SNPs (rs17035311, rs11729069, rs7661349, rs1007915, and rs62331150) with a minor allelic frequency (MAF) > 0.2 in Hans were chosen for testing. Three SNPs, rs17035311, rs7661349, and rs62331150, were analyzed because they were not involved in a linked pair (rs17035311 vs. rs11729069 and rs7661349 vs. rs1007915) (Figure 2). However, no statistically significant correlations were observed for any of the tested SNPs (Supplementary Table S1).

### 3.5. Methylation Rates of TET2

We examined 40 CpG sites in the *TET2* promoter. Compared with healthy donors, the SLE patients had significantly different methylation rates at two sites, *TET2* 105146116 and 105146718 (*p* = 0.048 and 0.040, respectively, as shown in Table 5). In SLE patients, the methylation rates were higher at *TET2* 105146116 but lower at *TET2* 105146718 than in the healthy donors. In region 105146072-154 and 105146218-331, there were even more significant differences in methylation rates (the examined CpG sites in the regions that had significant differences in methylation rates between SLE and healthy donors are shown in Supplementary Table S2) We then performed multivariate linear regression to analyze whether the methylation level in the CpG sites and regions with significance were influenced by age, gender, and medication (Table 6). However, the analysis demonstrated that the methylation level of CpGs was not driven by these variables.

### 3.6. The Relationships of SLE Disease Status and Global Methylation, Hydroxymethylation, and TET2 Methylation

We tried to directly link the relationships of TET2 methylation and global methylation or global hydroxymethylation in SLE, but no significant correlation was shown. In addition, we tested whether the SLE disease status or global methylation level was affected by TET2 methylation by causal inference analysis, but no significance was denoted (Supplementary Tables S3 and S4).

## 4. Discussion

In this study, we showed that altered methylation of *TET2* is involved in the dynamics of 5-mC and 5-hmC, contributing to the alteration of global DNA-methylation patterns in SLE PBMCs. Moreover, TET2 mRNA levels are positively associated with anti-dsDNA and negatively associated with complement levels, a proxy for the disease activity of SLE. The differences in global methylation, hydroxymethylation, *TET2* mRNA expression, and methylation rates in *TET2* CpG sites or regions between SLE the healthy donors were found to be independent of age, gender, and medication (methotrexate and cyclophosphamide).

Prior studies have indicated that the significant hypomethylation of interferon-regulated genes in T cells plays a pivotal role in SLE pathogenesis. Hypomethylated interferon-regulated genes are present in patients during flares and remission, suggesting that it is an early and persistent phenomenon in SLE [42,43]. In the findings of Mok et al., hypomethylation of HIF-related genes *IFI44* and *PRR4* was found to be associated with lupus nephritis [44], and the hypomethylated IL10 and IL1R2 genes were associated with disease activity [11]. In summary, genome-wide DNA-methylation studies have found that a decrease in the DNA-methylation level of genes is associated with SLE susceptibility [9], which is compatible with the global hypomethylation observed in the PBMCs of SLE patients in this study and our previous study [45].

There is emerging evidence showing that 5-hmC is an independent and epigenetic marker distinct from 5-mC and able to exert regulatory functions itself to influence gene transcription. In our study, we observed global hypohydroxymethylation in the PBMCs of SLE patients compared with healthy donors. Given the premise that DNA demethylation occurs through 5-mC dynamics, it has been observed that the depletion of hydroxymethylation-conferring TET proteins does not necessarily result in increased DNA methylation [29,46]. The correlation analysis of TET2 methylation and global methylation or hydroxymethylation failed to show a positive correlation, which implies that the 5-mc/5-hmc dynamics was not as straightforward as depicted in the chemical equation and that the role of TET2 methylation in the pathogenesis of SLE disease development must be far more complicated and yet to be fully elucidated. Zhao et al. identified 47 genes with decreased 5-hmC levels in their promoter regions in SLE CD4+ T cells and 2748 genes with increased 5-hmC levels in comparison with healthy donors [14]. Sui et al. reported that cyclin-dependent kinase inhibitor 1B (*CDKN1B*) exhibited significantly decreased levels of 5-hmC in the PBMCs of SLE patients, whereas three prime repair exonuclease (*TREX1*) and *CDKN1A* showed increased levels of 5-hmC [47]. The differential patterns of hydroxymethylation involving immune-related cells or key cytokine genes in the pathway leading to inflammation contributes to SLE pathogenesis. However, whether this is due to over- or underexpression of 5-hmC depends on the context, and how 5-hmC exerts itself to switch genes on or off has yet to be described.

Studies in human TET proteases have shown that mutations in *TET2* are most relevant to developing hematological malignancies [32,33,34] and are also associated with increased risks of autoimmunity in myelodysplastic syndrome patients [35]. TET2 and TET3 are expressed at higher levels than TET1 in peripheral T cells, and TET3 is believed to compensate for the loss of function of TET2 [48]. Our findings are consistent with this, wherein TET2 and TET3 were observed to be dominantly expressed in both SLE and healthy donors. Recently, it was found that TET2 masters important regulatory functions in the differentiation of T cells [49,50,51]. By affecting active DNA demethylation, it is possible to precisely regulate the maturation and expression of the signature cytokines of Th1 and Th17 [49]. SLE has previously been considered a “B-cell disease” due to the variety of autoantibodies produced by B cells as the culprits in SLE pathophysiology. However, studies are increasingly showing that T-cell dysfunction plays a more decisive role in the pathogenesis of SLE, including abnormal hyperactivity of Th17, changes in T-cell receptors, abnormalities in the related signaling pathways, and the dysfunction of T cells that regulate the ability to suppress inflammation [52]. This study used PBMCs, which are a heterogeneous mixture of mononuclear cells, as the experimental material, and T cells were the main components. *TET2* gene expression was significantly increased in SLE in this study, indicating that *TET2* may be involved in the pathogenesis of SLE by affecting DNA demethylation in T cells.

Clinically, serum levels of anti-dsDNA and complement concentrations (C3 and C4) are used as proxies to monitor disease activation and serve as one of the parameters of treatment response, which is supported by previous studies [41]. In this study, we found that the levels of *TET2* mRNA were also significantly correlated with those of anti-dsDNA, C3, and C4. *TET2* may have regulatory capabilities that affect overall immune function. However, more in-depth research is required to clarify whether the increase in *TET2* expression initiates or occurs in response to disease activity.

Although we observed that *TET2* mRNA expression varies in SLE patients, we assume that this was due to phenotypic variance. Three *TET2* SNPs were analyzed, and no statistically significant associations with disease were found. Meta-analysis of genome-wide associations for SLE have previously resulted in the identification of genetic variants in TET3 (rs6705628, rs4852324, and rs10207954) as susceptibility loci in Asian populations [53,54].

In our study, we demonstrated that, in SLE, the methylation rates were significantly lower in *TET2* Chr 4: 105146218-718 and significantly higher in Chr 4: 105146072-154. In Chr 4: 105146218-718, several transcriptional activators involving the noncanonical NF-kappa-B pathway were noted, such as DPF2 and RELB [55]. Dysregulation of noncanonical NF-κB activation affects various immune cells to different extents, such as (i) causing aberrant survival of self-reactive B cells, rendering them resistant to negative selection and leading to autoimmune antibody production associated with a number of autoimmune diseases, and (ii) resulting in aberrant chemokine production and inflammatory-cell recruitment in endothelial cells, T cells, and monocytes by diverse mechanisms [55]. In Chr 4: 105146116-154, where there is hypermethylation of CpG sites, there is also a binding site for the repressive transcription factor, enhancer of zeste homolog 2 (EZH2), which might play a key role in the T-cell epigenetic conformational changes that are associated with disease flare in lupus [51]. EZH2 reduces H3K27 trimethylation by the PI3K/AKT pathway, resulting in decreased binding between EZH2 and H3K27 [56,57]. In somatic-cell types and cancer-cell lines, H3K27me3 overlaps extensively with DNA methylation [58]. Hypermethylated regions correlated with higher disease activity of SLE were found to be enriched in binding sites for the repressive transcription factor EZH2 [51]. Consequently, downstream *TET2* genes might become transcriptionally activated from a state of inhibition, resulting in increased levels of *TET2* mRNA expression.

There are limitations to the study. First, our study used an ELISA technique to analyze the difference in methylation and hydroxymethylation. The technique does not allow differences to be shown in individual genes that may not be reflected in global changes. Second, although T lymphocytes are the majority, PBMC contains a variety of mononuclear cells, which could affect DNA methylation as an intrinsic factor, whereas DNA-methylation patterns are known to be tissue/cell specific and affect disease-phenotype differences. Finally, in a cross-sectional study like ours, only association and not the causal relationship between global methylation, global hydroxymethylation, and methylation level of TET2 is denoted.

## 5. Conclusions

In conclusion, this study delineated global hypomethylation and hypohydroxymethylation in the PBMCs of SLE patients and demonstrated, for the first time, that methylation of the *TET2* gene during SLE development and its mRNA expression are correlated with disease activity.

## Figures and Tables

**Table 1:** Demographic data of systemic lupus erythematosus and healthy donors.

	Female			Male		
	SLE (*n* = 91)	Health Donors (*n* = 76)	*p*	SLE (*n* = 10)	Health Donors (*n* = 21)	*p*
Age (mean ± SD)	38.34 ± 11.14	35.64 ± 10.24	0.108	34.3 ± 15.9	33.4 ± 15.17	0.878
<20	2	0		2	0	
20–30	22	24		3	3	
30–40	24	25		2	12	
40–50	27	24		1	3	
>50	16	3		2	2	
Drugs						
Methotrexate	4	N/A		2	N/A	
Cyclophosphamide	23	N/A		3	N/A	

SD, standard deviation. SLE, systemic lupus erythematosus. N/A, not applicable. *p*-values were obtained using *t*-tests.

**Table 2:** Global methylation and hydroxymethylation in the PBMCs of systemic lupus erythematosus patients and healthy donors.

	SLE (*n* = 101)	Healthy Donors (*n* = 100)	*p* ^a^
5-mC (%, mean ± SD)	1.109 ± 0.710	1.365 ± 0.830	0.021
5-hmC (%, mean ± SD)	0.063 ± 0.097	0.109 ± 0.133	0.012

SLE, systemic lupus erythematosus; 5-mC, 5-methylcytosine; 5-hmC, 5-hydroxymethylcytosine; SD, standard deviation. *p*-values were obtained using *t*-tests. ^a^ *p* < 0.05.

**Table 3:** Multivariate linear regression of factors related to alteration of methylation pattern.

	5-mC		5-hmC		*TET2* mRNA	
	β	*p*	β	*p*	β	*p*
Age	−0.150	0.142	−0.131	0.379	0.034	0.737
Gender	0.123	0.903	0.052	0.111	−0.027	0.795
Methotrexate	−0.176	0.086	−0.018	0.862	0.151	0.155
Cyclophosphamide	−0.112	0.270	0.144	0.163	0.118	0.253

β: β-coefficient; *p*: *p*-value.

**Table 4:** Correlation (r) between TET mRNA and disease activity of systemic lupus erythematosus, defined by levels of anti-dsDNA, C3, and C4.

mRNA	Anti-dsDNA	C3	C4
*TET1*	−0.096	0.119	−0.017
*TET2*	0.379 **	−0.257 *	−0.328 **
*TET3*	0.018	−0.179	−0.185

* *p* < 0.05; ** *p* < 0.01; correlation (r) values were obtained using Spearman’s correlation tests. Scatter plots shown in Supplementary Materials.

**Table 5:** Methylation rates at various CpG sites of *TET2* promoters.

CpG	SLE (%)	Healthy Donor (%)	*p* ^a^
105146116	1.222 ± 1.237	0.712 ± 0.646	0.048
105146072-154	4.738 ± 2.533	3.310 ± 1.207	0.006
105146218-331	1.242 ± 0.562	1.644 ± 0.649	0.014
105146718	0.526 ± 0.288	0.736 ± 0.463	0.040

SLE: systemic lupus erythematosus. ^a^ *p*-value was obtained by *t*-test.

**Table 6:** Multivariate regression of factors relevant to *TET2*-promoter methylation rates.

	105146116		105146072-154		105146218-331		105146718	
	β	*p*	β	*p*	β	*p*	β	*p*
Age	−0.294	0.051	−0.888	0.379	−0.261	0.317	−0.181	0.424
Gender	−0.251	0.091	−0.244	0.111	−0.410	0.147	−0.180	0.443
Methotrexate	−0.235	0.110	N/A	N/A	0.028	0.919	−0.072	0.743
Cyclophosphamide	−0.046	0.752	0.030	0.844	0.221	0.482	0.141	0.536

β: β-coefficient ; *p*: *p*-value; N/A: not applicable.

## Data Availability

Not applicable.

## Supplementary Materials

The following supporting information can be downloaded at: https://www.mdpi.com/article/10.3390/diagnostics12123006/s1, Table S1. Polymorphism of TETs in systemic lupus erythematosus and the controls; Table S2. CpG sites contained in the regions which have significant difference in methylation levels between SLE and health donors; Table S3. The correlation of TET2 methylation level and global methylation or global hydroxymethylation; Table S4. The interaction of TET2 methylation and SLE disease status. Figure S1. Correlation(r) between TETs mRNA and disease activity of systemic lupus erythematosus, defined by levels of anti-dsDNA, C3 and C4.
