# Supplementary material for: Methylation of TET2 Promoter Is Associated with Global Hypomethylation and Hypohydroxymethylation in Peripheral Blood Mononuclear Cells of Systemic Lupus Erythematosus Patients
Source: Diagnostics (Basel). 2022 Dec 1;12(12):3006. doi: 10.3390/diagnostics12123006 (PMC9776498; doi:10.3390/diagnostics12123006)
Supplement: Supplementary file 1 [file diagnostics-12-03006-s001.zip › diagnostics-1909049-supplementary.pdf]

Table S1. Polymorphism of TETs in systemic lupus erythematosus and the controls

| Gene SNPs | Genotype | SLE | Ctrl | p | Odds ratio (95% CI) | model | p | Odds ratio (95% CI) |
|---|---|---|---|---|---|---|---|---|
| TET2 rs17035311 (promoter -1157A>C) | AA | 68(60.71%) | 94(64.83%) | | | D | 0.50 | 1.19 (0.72-1.99) |
| | AC | 39(34.82%) | 47(32.41%) | 0.42 | 1.15 (0.68-1.94) | R | 0.46 | 1.65 (0.43-6.28) |
| | CC | 5(4.46%) | 4(2.76%) | 0.61 | 1.73 (0.45-6.67) | A | 0.42 | 1.20 (0.78-1.84) |
| TET2 rs7661349 (promoter -468 C>T) | CC | 73(65.18%) | 108(71.52%) | | | D | 0.27 | 1.34 (0.79-2.27) |
| | CT | 37(33.01%) | 40(26.49%) | 0.25 | 1.37 (0.80-2.34) | R | 0.91 | 0.90 (0.15-5.46) |
| | TT | 2(1.79%) | 3(1.99%) | 0.99 | 0.99 (0.16-6.05) | A | 0.35 | 1.25 (0.79-1.98) |
| TET2 rs62331150 (INTRON +1564 G>T) | CC | 73(65.18%) | 108(71.52%) | | | D | 0.75 | 1.34 (0.79-2.27) |
| | CT | 37(33.01%) | 40(26.49%) | 0.25 | 1.37 (0.80-2.34) | R | 0.057 | 0.90 (0.15-5.46) |
| | TT | 2(1.79%) | 3(1.99%) | 0.99 | 0.99 (0.16-6.05) | A | 0.12 | 1.25 (0.79-1.98) |

Figure S1. Correlation(r) between TETs mRNA and disease activity of systemic lupus erythematosus, defined by levels of anti-dsDNA, C3 and C4

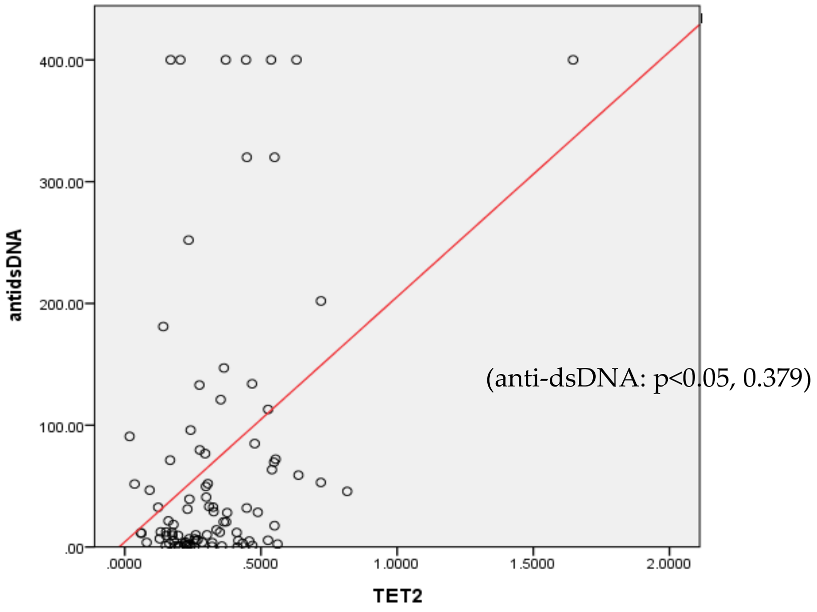


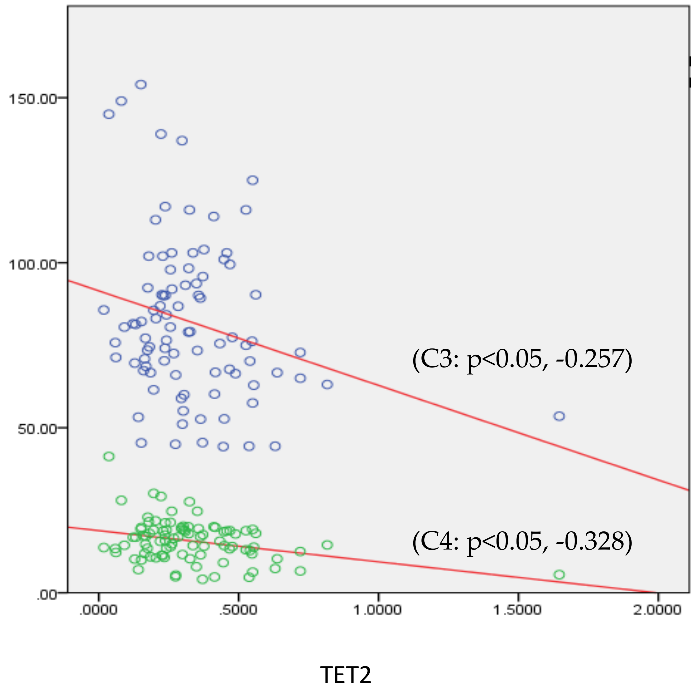

Table S2. CpG sites contained in the regions which have significant difference in methylation levels between SLE and health donors

| CpG sites | SLE (%) | Health donor (%) |
|---|---|---|
| 105146072 | 0.9896±1.1803 | 0.7843±0.4181 |
| 105146103 | 1.2118±1.0724 | 0.9889±0.6971 |
| 105146116 | 1.2220±1.2369 | 0.7209±0.6377 |
| 105146142 | 0.6409±0.9947 | 0.5513±0.7484 |
| 105146154 | 0.5676±1.0086 | 0.2638±0.4219 |
| 105146218 | 0.5316±0.3592 | 0.7725±0.4517 |
| 105146331 | 0.6916±0.4985 | 0.8608±0.4809 |

Table S3. The correlation of TET2 methylation level and global methylation or global hydroxymethylation

| CpG | TET2 methylation and global methylation, p | TET2 methylation and global hyroxymethylation, p |
|---|---|---|
| 105146116 | 0.179 | 0.258 |
| 105146072-154 | 0.899 | 0.513 |
| 105146218-331 | 0.243 | 0.641 |
| 105146718 | 0.596 | 0.473 |

p, p-value. p-values were obtained using Pearson's correlation.

Table S4. The interaction of TET2 methylation and SLE disease status

| CpG | TET2 methylation → global methylation → SLE, p | TET2 methylation → global hydroxymethylation → SLE, p |
|---|---|---|
| 105146116 | 0.828 | 0.804 |
| 105146072-154 | 0.855 | 0.897 |
| 105146218-331 | 0.862 | 0.827 |
| 105146718 | 0.632 | 0.895 |

p, p-value. p-values were obtained using causal inference analysis.
